# Solitaire Thrombectomy for Acute Stroke Due to Intracranial Atherosclerosis-Related Occlusion: ROSE ASSIST Study

**DOI:** 10.3389/fneur.2018.01064

**Published:** 2018-12-11

**Authors:** Jin Soo Lee, Seong-Joon Lee, Ji Man Hong, Jin Wook Choi, Joonsang Yoo, Jeong-Ho Hong, Chang-Hyun Kim, Yong-Won Kim, Dong-Hun Kang, Yong-Sun Kim, Yang-Ha Hwang, Sung-Il Sohn

**Affiliations:** ^1^Department of Neurology, Ajou University School of Medicine, Ajou University Medical Center, Suwon, South Korea; ^2^Department of Radiology, Ajou University School of Medicine, Ajou University Medical Center, Suwon, South Korea; ^3^Department of Neurology, Keimyung University Dongsan Medical Center, Daegu, South Korea; ^4^Department of Neurosurgery, Keimyung University Dongsan Medical Center, Daegu, South Korea; ^5^Department of Neurology, Kyungpook National University School of Medicine, Daegu, South Korea; ^6^Department of Radiology, Kyungpook National University School of Medicine, Daegu, South Korea; ^7^Department of Neurosurgery, Kyungpook National University School of Medicine, Daegu, South Korea

**Keywords:** cerebral infarction, stent, thrombectomy, intracranial atherosclerosis, intracranial embolism

## Abstract

**Background:** Solitaire, a representative stent retriever, has shown high performance in removing embolic clots. However, its reperfusion potential in intracranial atherosclerotic stenosis (ICAS)-related occlusions has rarely been reported. In this ROSE ASSIST study, we hypothesized that Solitaire device is as effective and safe for removing *in situ* thrombi in ICAS-related occlusions as it is for removal of embolic occlusions.

**Methods:** Data from ASIAN KR, an observational multicenter registry (*n* = 720) enrolling patients who have undergone endovascular treatment for acute cervicocephalic artery occlusions, were retrospectively reviewed. Through blinded evaluations, occlusions were classified as ICAS-related (significant fixed focal stenosis observed at the occlusion site during endovascular treatment) or embolic (no or minimal stenosis observed). Among patients treated within 720 min after stroke onset, those who undertook Solitaire thrombectomy and whose underlying etiology was ICAS-related or embolic were included. The primary endpoint was immediate successful reperfusion (modified Treatment In Cerebral Ischemia 2b−3) after Solitaire stent retrieval. The safety endpoint included intracerebral hemorrhagic transformation and subarachnoid hemorrhage. Comparative analyses were performed between embolic and ICAS-related occlusions with 2:1 propensity score matching.

**Results:** In total, 303 patients (embolic, 228; ICAS-related, 75) were included in the analyses. As for the primary endpoint, the immediate successful reperfusion rate following Solitaire thrombectomy did not differ between the two etiologic groups after propensity score matching (73.1% embolic vs. 65.8% ICAS-related, *p* = 0.261). The final successful reperfusion grade was also similar in the two groups (79.3 vs. 72.0%, *p* = 0.219). The grades and frequencies of intracerebral hemorrhagic transformation and subarachnoid hemorrhage did not differ between groups (*p* = 0.134 and *p* = 0.269, respectively).

**Conclusions:** The immediate reperfusion performance in terms of thrombus removal of Solitaire thrombectomy for ICAS-related occlusions was similar to that for embolic occlusions.

## Introduction

Mechanical thrombectomy with stent retrievers has achieved high level of evidence for the treatment for patients with acute ischemic stroke caused by intracranial large artery occlusion (1). The mechanical thrombectomy method utilizing stent retrievers results in both a high recanalization rate and a good prognosis in the embolic occlusion cases (2–5). As for a prototype stent retriever, the Solitaire device, its efficacy is well-known both in Asian (6–8) and Western (9, 10) countries. However, its effectiveness for large artery occlusions due to intracranial atherosclerotic stenosis (ICAS) and *in situ* thrombosis has rarely been reported.

In North America and Europe, acute cerebral infarctions from intracranial large artery occlusions are most often due to embolism. In contrast, in Asia, acute ischemic strokes from intracranial large artery occlusions are often caused by *in situ* atherosclerotic mechanisms (11–13). The frequency of ICAS-related occlusions is reported as 15.2% of intracranial large artery occlusions involving the anterior circulation (13) and as 37.5% of occlusions involving the posterior circulation (14). A French study reported that it accounted for only 5.5% of patients with stent retrieval treatment (15).

To date, the performance of stent retrievers is mostly proven for embolic occlusions. In a preliminary study, the Solitaire stent was efficient in removing *in situ* thrombi in ICAS-related occlusions (16). Additionally, Solitaire stent thrombectomy achieved immediate successful reperfusion in several consecutive patients. In this Role of Solitaire in Endovascular Treatment for Acute Serious Stroke Due to Intracranial *in situ* Thrombosis (ROSE ASSIST) study, we hypothesized that Solitaire thrombectomy is as effective and safe for thrombus removal in ICAS-related occlusions as it is for embolic occlusions and analyzed in a large retrospective registry.

## Methods

### The ASIAN KR Registry and the ROSE ASSIST Study

The ASIAN KR registry was created for observational research and consists of consecutive patients, aged 18 years or older, who received endovascular treatment (EVT) for the treatment of acute ischemic stroke due to intracranial and/or extracranial large vessel occlusion (17). The type of EVT procedure was chosen at the discretion of the treating physician. Both de-identification and the allocation of study identification numbers were performed for all clinical data. After de-identification and blinding of clinical data, core laboratory imaging analyses were performed to ensure consistent grading and to eliminate bias. The data collection protocol was approved by the institutional review board of each respective hospital and implemented in accordance with the ethical standards of the 1964 Declaration of Helsinki and its later amendments.

The ROSE ASSIST study was designed for proving effectiveness and safety of Solitaire thrombectomy for thrombus removal in ICAS-related occlusions. Although being sponsored by Medtronic, the study was conducted independently from the company.

### Etiologic Classification of Target Occlusive Lesions

The etiology of target large vessel occlusion was determined by stepwise angiographic analysis with procedural digital subtraction images (YHH, LJS) and postprocedural repeat angiographies obtained during admission (JSY) (14, 18–20). First, uncommon occlusive etiologies including dissection, Moyamoya disease and vasculitis, and the extracranial artery disease-related occlusions were excluded. Second, when the occluded vessel was completely recanalized, the etiology was classified as embolic occlusion. Third, remaining stenosis >70% or less-degree stenosis with a tendency of re-occlusion or flow impairment during the procedure and on final angiography was classified as ICAS-related occlusion. When recanalization could not be achieved during the procedure to determine the etiology, it was specifically classified as intractable occlusion cases, which were also excluded from the current study. In most cases, the classification of ICAS-related and embolic occlusion was not difficult by angiographic evaluations. However, for some cases, including mild stenosis, we determined the underlying etiology with consensus between YHH and JSL. Lastly, this mechanism was further evaluated by repeat angiography following EVT during admission (JSY).

### Inclusion and Exclusion Criteria

A. Inclusion criteria:
  - Patients with acute ischemic stroke who had intracranial large artery occlusion (arterial occlusive lesion grade 0) on baseline computed tomography (CT) or magnetic resonance angiography within the vascular territory corresponding to the neurologic deficit observed at the neurologic examination.
  - Time from stroke onset to presentation < 12 h.
  - Patients who underwent the endovascular revascularization treatment with the Solitaire stent.
B. Exclusion criteria:
  - Intracranial large artery occlusion due to uncommon stroke etiology including arterial dissection, Moyamoya disease, and vasculitis.
  - Tandem intracranial large artery occlusions caused by extracranial arterial disease.
  - Patients in whom the arterial lesion status could not be reliably assessed due to either persistent occlusion or incomplete recanalization.

### Propensity Score Matching

To reduce the effects of selection bias and potential confounding between the two groups, we performed adjustments for significant differences in the baseline characteristics of the patients using propensity score matching. The propensity scores were estimated using multiple logistic-regression analysis. Variables chosen for inclusion in the model were age, sex, intravenous recombinant tissue plasminogen activator use, onset-to-puncture time, intracranial occlusion locations, and Solitaire use as primary vs. rescue treatment. After estimating the propensity score, the ICAS and embolic groups were matched at a ratio of one to two. This matching was performed using R version 3.4.1 with MatchIt package (version 3.0.2).

### Endpoints

The primary endpoint was immediate successful reperfusion (modified Treatment In Cerebral Ischemia 2b−3) after Solitaire stent thrombectomy. The secondary safety endpoints included (1) degree and frequency of intracerebral hemorrhage on non-contrast CT or magnetic resonance imaging within 7 days, and (2) degree and frequency of subarachnoid hemorrhage on non-contrast CT within 24 h after EVT. Intracerebral hemorrhages were classified in accordance with criteria defined by the European Cooperative Acute Stroke Study (S.I.S.) (21). Subarachnoid hemorrhage was classified according to the modified Fisher scale (S.I.S.) (22).

### Statistics

Differences between the groups were analyzed using χ^2^ tests for categorical variables, the Mann-Whitney test for ordinal variables and Student's *t*-tests for continuous variables. After propensity score matching, independent statistics were also used due to incomplete pairing. For evaluating procedural outcomes on the primary use of Solitaire thrombectomy, variables were compared in embolic and ICAS groups without propensity score matching. *P* < 0.05 were considered significant. Statistical analysis was performed using the SPSS statistical package (version 22.0, Chicago, IL).

## Results

### Baseline Characteristics and Propensity Score Matching

**Figure 1:**
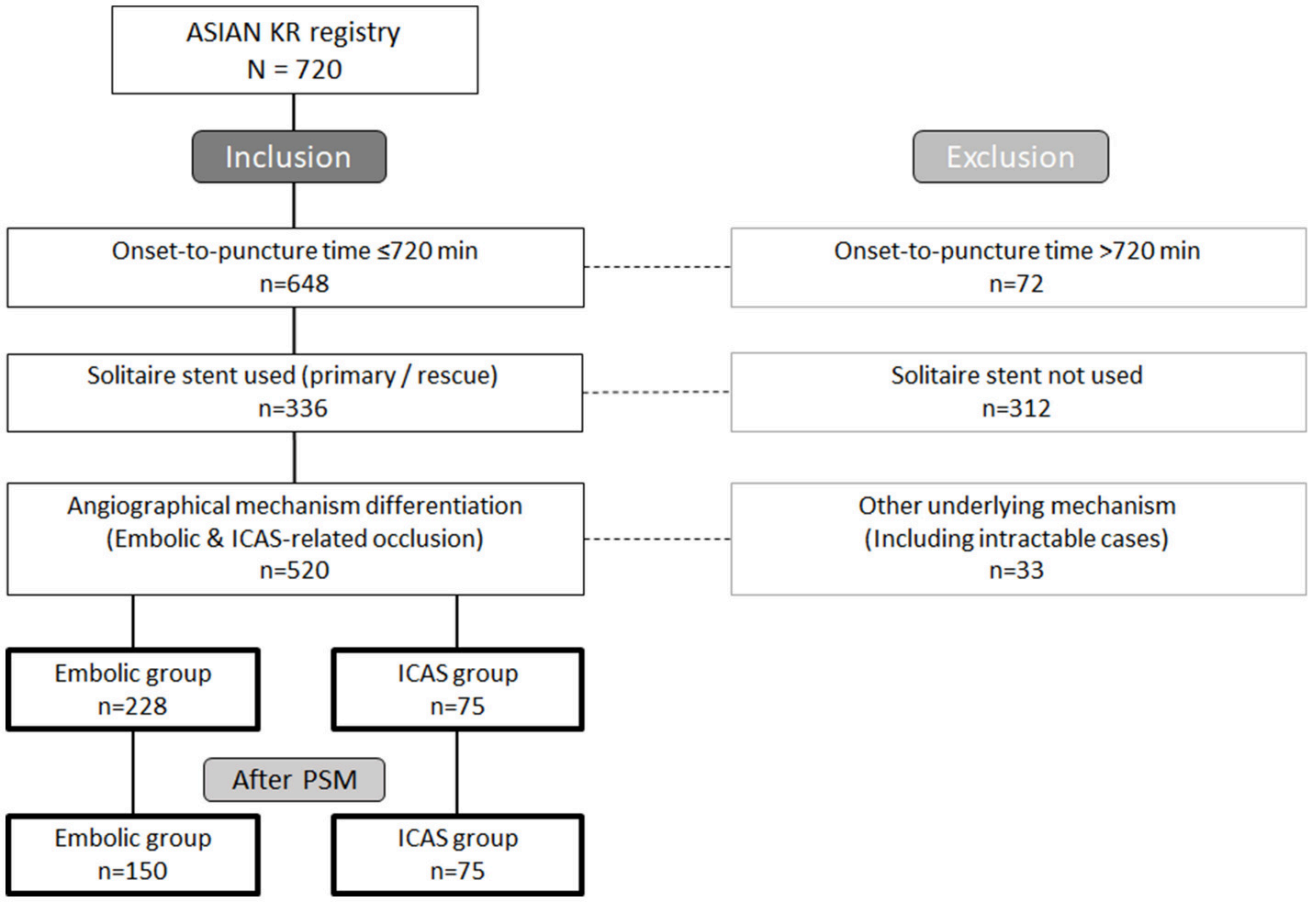
Flowchart illustrating the current study. PSM, propensity score matching.

Seventy-five and 228 patients were included in the ICAS and embolic groups, respectively, based on the study criteria (Figure 1). The baseline characteristics and treatments are summarized in Table 1. Compared to those in the embolic group, patients in the ICAS group were younger (68.5 ± 13.4 vs. 64.3 ± 14.0 years, *p* = 0.019) and presented higher total cholesterol levels (163 ± 38 vs. 183 ± 41 mg/dL, *p* < 0.001), low-density lipoprotein levels (95 ± 34 vs. 115 ± 38 mg/dL, *p* < 0.001), and systolic (142 ± 26 vs. 149 ± 26 mmHg, *p* = 0.034) and diastolic (81 ± 15 vs. 85 ± 14 mmHg, *p* = 0.040) blood pressure. They were also more frequently male (47.8 vs. 64.0%, *p* = 0.015), more commonly smokers (16.2 vs. 32.0%, *p* = 0.003), and more frequently presented occlusions in the M1 portion of the middle cerebral artery (MCA) (*p* < 0.001). They also less commonly had atrial fibrillation as a comorbidity (59.6% vs. 22.7%, *p* < 0.001). When compared to those in the embolic group, patients in the ICAS group underwent intravenous recombinant tissue plasminogen activator treatment less frequently (64.9% vs. 44.0%, *p* = 0.001) and had significantly longer onset-to-puncture times (258 ± 132 min vs. 333 ± 164 min, *p* < 0.001).

**Table 1:** Baseline characteristics and treatments used before and after propensity score matching.

	**Before propensity score matching**			**After propensity score matching**		
	**Embolic**	**ICAS**	***P***	**Embolic**	**ICAS**	***p***
Number	228	75		150	75	
Age, years	68.5 ± 13.4	64.3 ± 14.0	0.019	64.9 ± 14.0	64.3 ± 14.0	0.754
Male sex	109 (47.8%)	48 (64.0%)	0.015	90 (60.0%)	48 (64.0%)	0.561
Premorbid mRS, median [IQR]	0 [0–0]	0 [0–0]	0.880	0 [0–0]	0 [0–0]	0.695
Initial NIHSS score, median [IQR]	17 [13–20]	16 [12–20]	0.316	16.5 [13–20]	16 [12–20]	0.574
Baseline occlusion location			< 0.001			< 0.001
ICA T	94 (41.2%)	15 (20.0%)		63 (42.0%)	15 (20.0%)	
MCA M1	94 (41.2%)	46 (61.3%)		60 (40.0%)	46 (61.3%)	
MCA M2	23 (10.1%)	1 (1.3%)		15 (10.0%)	1 (1.3%)	
VBA	14 (6.1%)	13 (17.3%)		9 (6.0%)	13 (17.3%)	
Others	3 (1.3%)	0 (0%)		3 (2.0%)	0 (0%)	
Hypertension	142 (62.3%)	49 (65.3%)	0.635	89 (59.3%)	49 (65.3%)	0.384
Diabetes mellitus	47 (20.6%)	22 (29.3%)	0.118	31 (20.7%)	22 (29.3%)	0.149
Atrial fibrillation	136 (59.6%)	17 (22.7%)	< 0.001	85 (56.7%)	17 (22.7%)	< 0.001
Coronary artery occlusive disease	29 (12.7%)	6 (8.0%)	0.267	16 (10.7%)	6 (8.0%)	0.526
Smoking	37 (16.2%)	24 (32.0%)	0.003	30 (20.0%)	24 (32.0%)	0.047
Admission glucose level, mg/dl	136 ± 47	151 ± 63	0.058	132 ± 46	151 ± 63	0.021
Total cholesterol level, mg/dl	163 ± 38	183 ± 41	< 0.001	163 ± 39	183 ± 41	0.001
Triglyceride level, mg/dl	110 ± 63	135 ± 141	0.136	116 ± 64	135 ± 141	0.180
High-density lipid level, mg/dl	48 ± 27	45 ± 10	0.357	49 ± 32	45 ± 10	0.349
Low-density lipid level, mg/dl	95 ± 34	115 ± 38	< 0.001	95 ± 37	115 ± 38	< 0.001
C-reactive protein level	0.74 ± 1.96	0.81 ± 1.96	0.775	0.74 ± 2.17	0.81 ± 1.96	0.811
Initial systolic blood pressure, mmHg	142 ± 26	149 ± 26	0.034	143 ± 27	149 ± 26	0.096
Initial diastolic blood pressure, mmHg	81 ± 15	85 ± 14	0.040	82 ± 15	85 ± 14	0.162
Intravenous thrombolysis	148 (64.9%)	33 (44.0%)	0.001	83 (55.3%)	33 (44.0%)	0.109
Onset to puncture time, min	258 ± 132	333 ± 164	< 0.001	296 ± 144	333 ± 164	0.080
Puncture to final angiography time, min	80 ± 48	90 ± 48	0.138	87 ± 52	90 ± 48	0.710
Solitaire use as			0.142			0.637
Primary treatment	134 (59.0%)	37 (49.3%)		79 (52.7%)	37 (49.3%)	
Rescue treatment	93 (41.0%)	38 (50.7%)		71 (47.3%)	38 (50.7%)	

*ICAS, intracranial atherosclerotic stenosis; mRS, modified Rankin Scale; NIHSS, national Institute of Health stroke scale; ICA, internal carotid artery; MCA, middle cerebral artery; VBA, vertebrobasilar artery*.

After matching, the differences in age, sex, intravenous recombinant tissue plasminogen activator treatment, and onset-to-puncture time between the two groups were balanced. The proportions of MCA M1 and vertebrobasilar artery occlusions, however, were still higher in the ICAS group, while internal carotid artery terminal and MCA M2 occlusions were more frequent in the embolic group (*p* < 0.001). Solitaire thrombectomy was used in 79 patients (52.7%) as primary treatment and in 71 (47.3%) as rescue treatment in the embolic group, whereas it was employed in 38 patients (49.3%) as primary treatment and in 38 (50.7%) as rescue treatment in the ICAS group (*p* = 0.637).

### Immediate Reperfusion Performance and Safety

**Figure 2:**
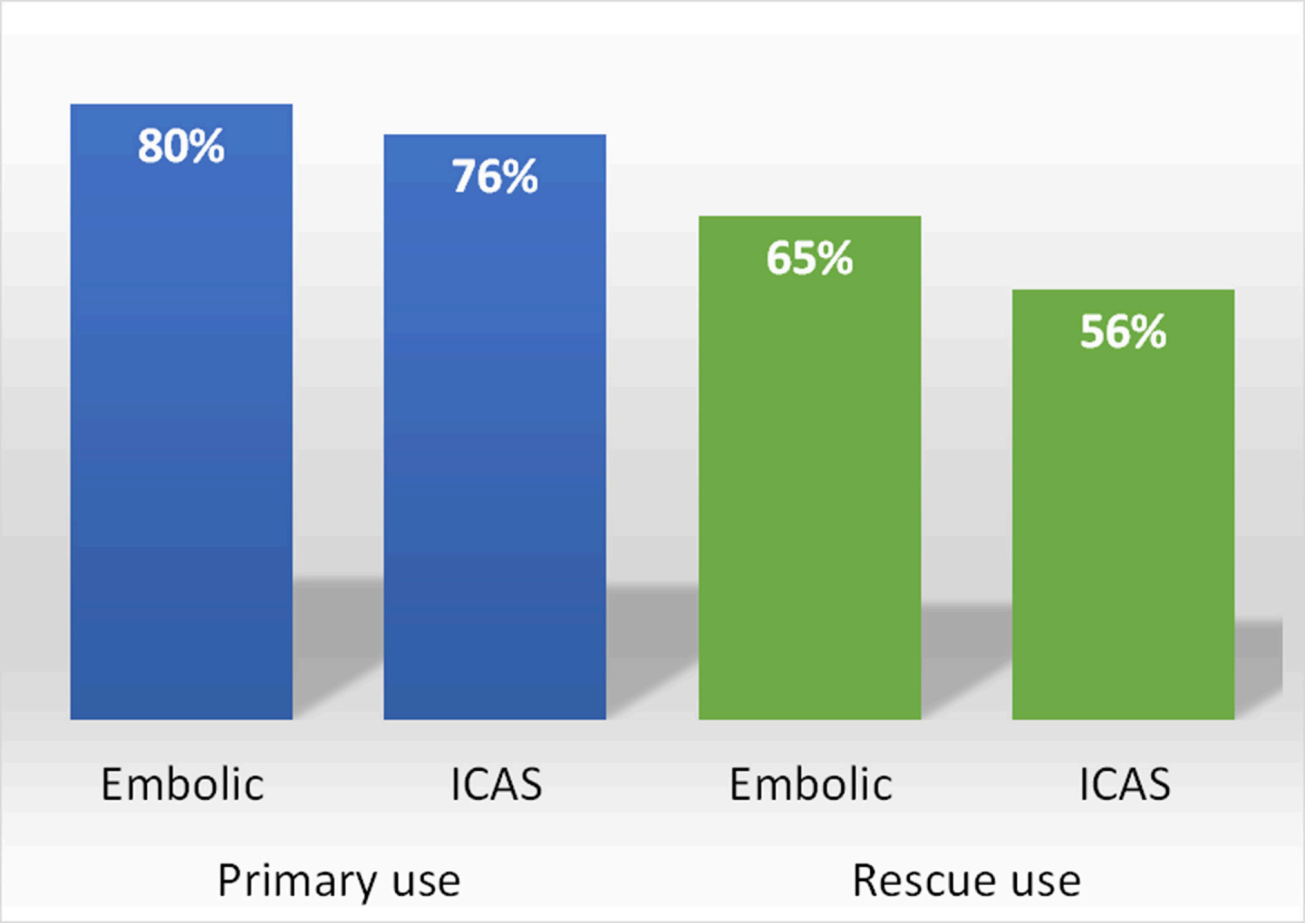
Immediate successful reperfusion rates in embolic and ICAS-related occlusions after Solitaire thrombectomy used as either primary or rescue treatment.

**Figure 3:**
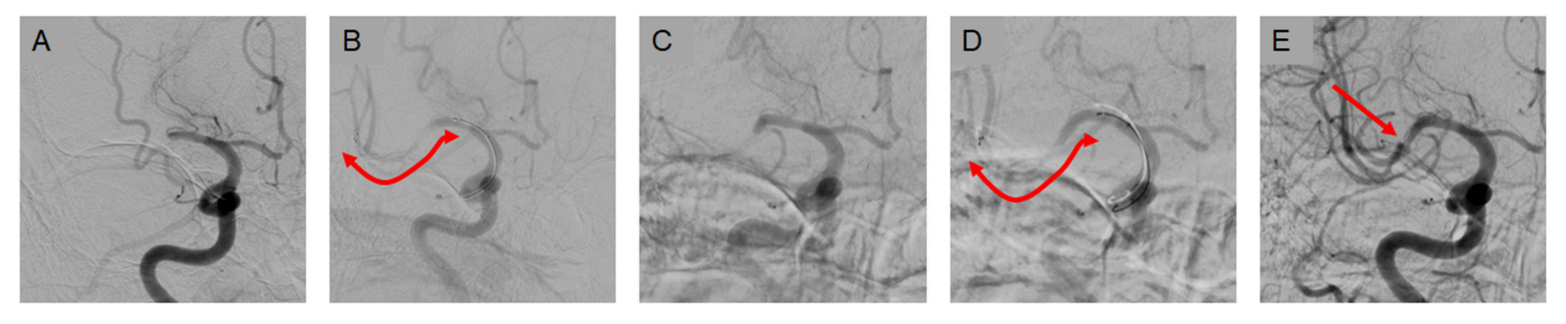
A case of primary use of Solitaire thrombectomy in ICAS-related occlusions. **(A)** Occlusion is seen in the middle M1 segment of the middle cerebral artery prior to endovascular treatment. **(B)** The Solitaire stent was deployed for thrombectomy as primary treatment. **(C)** After the first pass, the clot was removed and partial recanalization was achieved, although focal stenosis was observed. **(D,E)** After 2 more passes, complete reperfusion was achieved despite the remaining focal stenosis.

**Figure 4:**
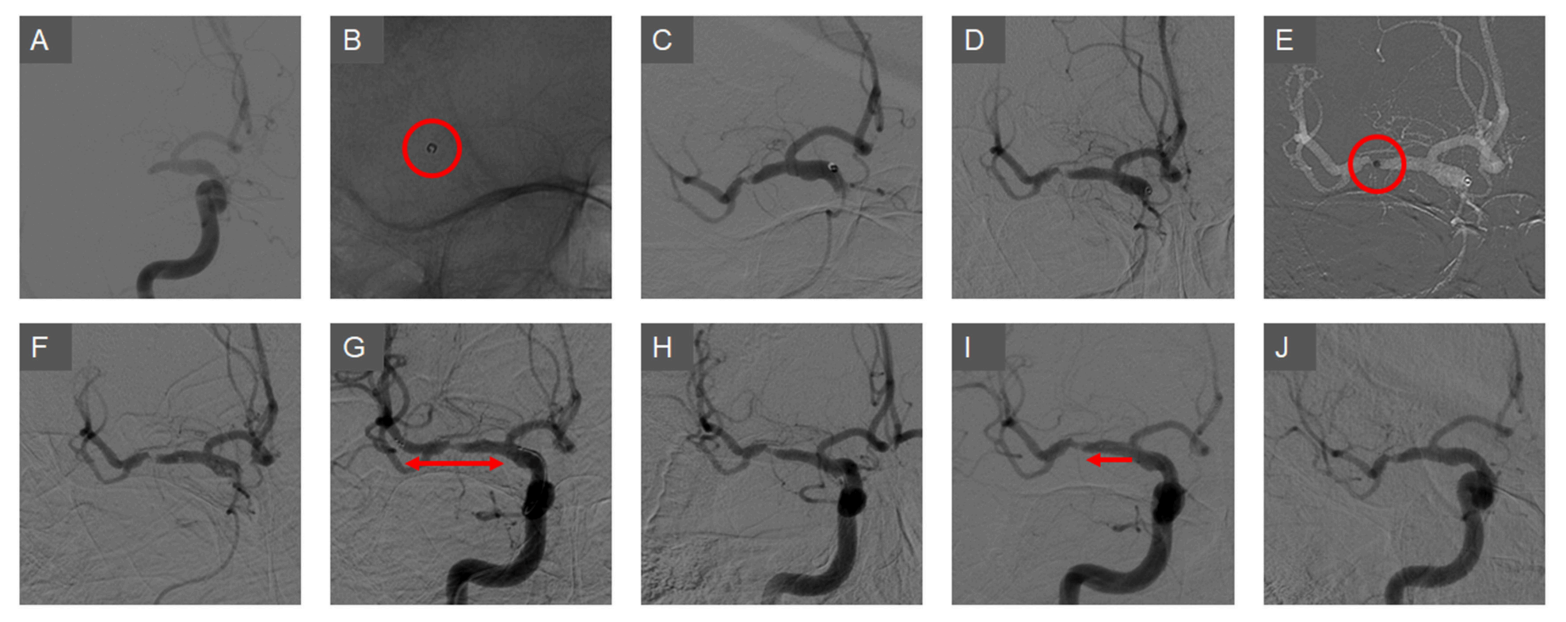
A case of rescue treatment with the Solitaire stent in ICAS-related occlusions. **(A)** A right M1 occlusion is seen prior to the endovascular treatment. **(B)** The contact aspiration technique was used as the primary endovascular treatment. **(C)** The clot was removed, however, the focal stenosis was observed in the segment. **(D)** The vascular lesion appeared to be reoccluded soon after the treatment. **(E)** The aspiration catheter was placed further into the lesion and manipulated. **(F)** The vascular lesion appeared more aggravated and blood flow appeared to be impaired. **(G)** The Solitaire stent was deployed as a rescue treatment method. **(H)** After one pass, the vascular lesion was slightly improved and blood flow was somewhat restored. **(I)** After two more passes, the vessel was further recanalized. To prevent re-occlusion, low-dose tirofiban was infused. **(J)** In the final angiography, the vessel lesion appeared more stable and reperfusion was successfully achieved.

The results of comparing outcomes are summarized in Table 2. The immediate successful reperfusion rate following Solitaire thrombectomy, which was the primary outcome, did not differ between the embolic and ICAS groups (73.1 vs. 65.8%, *p* = 0.261). Specifically, the immediate success rates were relatively higher in the ICAS-related occlusion cases when Solitaire thrombectomy was used as primary treatment (79.7 vs. 75.7%, *p* = 0.619) than in cases wherein it was used as rescue treatment (65.2 vs. 55.6%, *p* = 0.341) (Figure 2). Representative cases are illustrated in Figures 3, 4. Overall, EVT performed using all feasible methods resulted in similar final reperfusion rates in the two groups (embolic vs. ICAS, 79.3 vs. 72.0%, *p* = 0.219).

**Table 2:** Revascularization outcomes.

	**Before propensity score matching**			**After propensity score matching**		
	**Embolic**	**ICAS**	***P***	**Embolic**	**ICAS**	***p***
Immediate successful reperfusion by Solitaire	75.5%	65.8%	0.106	106 (73.1%)	48 (65.8%)	0.261
Final successful reperfusion	183 (80.3%)	54 (72.0%)	0.133	119 (79.3%)	54 (72.0%)	0.219
Post-procedural intracerebral hemorrhagic transformation			0.175			0.134
None	138 (60.5%)	56 (74.7%)		88 (58.7%)	56 (74.7%)	
Hemorrhagic transformation 1	18 (7.9%)	3 (4.0%)		10 (6.7%)	3 (4.0%)	
Hemorrhagic transformation 2	32 (14.0%)	6 (8.0%)		22 (14.7%)	6 (8.0%)	
Parenchymal hematoma 1	20 (8.8%)	3 (4.0%)		16 (10.7%)	3 (4.0%)	
Parenchymal hematoma 2	20 (8.8%)	7 (9.3%)		14 (9.3%)	7 (9.3%)	
Post-procedural subarachnoid hemorrhage			0.298			0.269
None	195 (85.5%)	70 (93.3%)		126 (84.0%)	70 (93.3%)	
Grade 1	15 (6.6%)	2 (2.7%)		10 (6.7%)	2 (2.7%)	
Grade 2	5 (2.2%)	0 (0%)		2 (1.3%)	0 (0%)	
Grade 3	4 (1.8%)	0 (0%)		4 (2.7%)	0 (0%)	
Grade 4	9 (3.9%)	3 (4.0%)		8 (5.3%)	3 (4.0%)	
Modified Rankin Scale 0–2 at 3 months	104 (45.8%)	28 (37.3%)	0.199	71 (47.3%)	28 (37.3%)	0.154
Mortality at 3 months	36 (15.9%)	12 (16.0%)	0.977	24 (16.0%)	12 (16.0%)	1.000

*ICAS, intracranial atherosclerotic stenosis; IQR, interquartile range*.

The degree and frequency of post-procedural intracerebral hemorrhage, which were the secondary (safety) endpoints, did not differ between the two groups (*p* = 0.134). In concordance, the degree and frequency of post-procedural subarachnoid hemorrhage did not differ between the groups (*p* = 0.269). Finally, also the rate of independent functional outcomes did not differ between the two groups (47.3 vs. 37.3%, *p* = 0.154).

### Subgroup Analyses for the Primary Use of Solitaire

After primary thrombectomy with Solitaire device, immediate side effects and rescue treatments were evaluated (Table 3). In terms of immediate side effects following primary Solitaire thrombectomy, compared to the embolic group, target vessel injury (3.7 vs. 13.5%, *p* = 0.025) and reocclusion (1.5 vs. 24.3%, < 0.001) more frequently occurred in the ICAS group whereas the occurrence of vasospasm and clot migration into another vessel did not differ. As for rescue treatments, tirofiban local infusion (7.5 vs. 40.5%, *p* < 0.001), intracranial balloon angioplasty (1.5 vs. 8.1%, *p* = 0.034) and intracranial stenting (3.7 vs. 16.2%, *p* = 0.006) were more frequently performed in the ICAS group. Procedural time was longer in the ICAS group (61.6 min vs. 79.6 min, *p* = 0.012).

**Table 3:** Procedural outcomes on the primary use of Solitaire thrombectomy (not matched).

	**Embolic**	**ICAS**	***P***
Number	134	37
**IMMEDIATE SIDE EFFECTS**			
Vasospasm	4 (3.0%)	2 (5.4%)	0.479
Clot migration into another vessel	6 (4.5%)	3 (8.1%)	0.381
Target vessel injury	5 (3.7%)	5 (13.5%)	0.025
Immediate reocclusion	2 (1.5%)	9 (24.3%)	< 0.001
**RESCUE TREATMENTS**			
Tirofiban local infusion	10 (7.5%)	15 (40.5%)	< 0.001
Intracranial balloon angioplasty	2 (1.5%)	3 (8.1%)	0.034
Intracranial stenting	5 (3.7%)	6 (16.2%)	0.006
Procedural time (min)	61.6 ± 33.0	79.6 ± 48.9	0.012

## Discussion

In the current study, we investigated whether mechanical thrombectomy using the Solitaire stent had an immediate reperfusion performance in ICAS-related occlusions comparable to that in usual embolic occlusions. To minimize mismatching of baseline characteristics, we performed propensity score matching (23). The immediate reperfusion performance of Solitaire thrombectomy was substantial for ICAS-related occlusions when compared to embolic occlusions. Moreover, it was safe as shown by both the subarachnoid hemorrhage and intracerebral hemorrhagic transformation frequencies. Nevertheless, in subgroup analysis, ICAS-related occlusions were more associated with target vessel injury and immediate reocclusion than embolic occlusions, which resulted in an overall longer procedure time than embolic occlusions as they required more caution.

It has previously been suggested that thrombus formation and propagation are important pathomechanisms of neurological symptoms and signs resulting from ICAS-related occlusions in acute ischemic stroke (19). Moreover, previous anecdotal study suggested that the Solitaire stent retriever could be used to remove a thrombus in the ICAS-related occlusion (16). Nevertheless, the aforementioned findings needed further exploration in a larger population-based study with comparative analyses in standard embolic occlusion cases with baseline variable adjustments. Therefore, our group collected and combined multicenter registry data and evaluated the images in each imaging core lab. Our final outcome measures indicate that the rate of thrombus removal, which was represented by immediate reperfusion following the use of the Solitaire stent, was high for ICAS-related occlusions but not significantly different compared to that in the embolic occlusion cases. More specifically, the immediate reperfusion performance in the ICAS-related occlusion cases was nearly the same as that observed for embolic occlusions, when the Solitaire was used as a primary treatment method (76 vs. 80%).

Some physicians may become concerned that stent retrieval may induce vessel injury at the stenotic site. The Solitaire stent, which has an overlapping design, is expected to be smoothly retrieved through the stenotic vessel. Overall, the use of Solitaire thrombectomy during EVT did not have different outcomes in terms of subarachnoid hemorrhage occurrence in patients with ICAS-related occlusion compared to those with embolic occlusion. Nevertheless, target vessel injury following the primary use of Solitaire thrombectomy occurred more frequently in ICAS-related occlusion than in embolic occlusion. However, the relatively high frequency of those immediate side effects does not halt the primary use of Solitaire stent because its success rate of immediate reperfusion on ICAS-related occlusion was similar to that of embolic occlusion.

Several endovascular thrombectomy modalities, differing in stent retrieval, can be used for the treatment of the ICAS-related occlusion. However, these modalities were not addressed in the current study. For example, the contact aspiration technique is commonly used as a secondary treatment in cases intractable with stent retrieval, or vice versa (24). However, the switch between the two major types of thrombectomy techniques when stenosis is observed during a primary thrombectomy may be no longer needed. In a very recent study, up to four passes of stent retrieval achieved around 95% of reperfusion success for all types of intracranial large artery occlusions (25). It is experienced that a focal fixed stenosis can be mostly found within a few passes of stent retrieval for ICAS-related occlusions. We plan to further study the effectiveness and immediate side effects, such as vessel injury resulting from stent-retrieval thrombectomy vs. contact aspiration, when such techniques are used as primary treatments for ICAS-related occlusion.

Immediate reocclusion more frequently occurred in ICAS-related occlusion than in embolic occlusion in our subgroup analysis during the primary use of Solitaire thrombectomy. Reocclusion is associated with stent-retrieval failure as explained by truncal-type occlusion (26). The reocclusion rate of 24.3% in the current study is somewhat less than that reported previously. This reocclusion rate can vary based on different definitions. In the current study, the occurrence of reocclusion was evaluated only after the primary Solitaire thrombectomy. However, most cases of ICAS-related occlusions needed rescue treatments; therefore, the rate of reocclusion in the current analysis was calculated in a short time between the end of primary thrombectomy and the start of next rescue treatment, likely resulting in a lower rate. A recent study reported that the reocclusion rate accounted for 57.1% in ICAS-related occlusion and it was defined as an event occurring within at least 20 min after a sufficient recanalization (27). Another previous study showed reocclusion occurred in 65% cases of *in situ* thromboocclusions, which can be considered as ICAS-related occlusions (28). This study also defined reocclusion as the event count after recanalization. Another study reported 40% of instant reocclusion, which counted only the events that occurred during procedure only in M1 occlusion population (18). The tendency of reocclusion may also differ among vascular beds. On the other hand, delayed reocclusions that occurred after the procedure, was reported in 8 out of 40 patients (20%) in the above study (18). Another study showed delayed reocclusion in 15.7% of ICAS-related occlusions on repeat angiography (20). For further studies, it would be interesting if these reocclusion rates were compared according to primary thrombectomy methods.

Rescue treatment should be performed based on reocclusion tendency or tight stenosis in ICAS-related occlusions (19, 29). Local infusion of an antiplatelet agent may be one option. Tirofiban is an example of antiplatelet treatment, which is a glycoprotein 2b/3a inhibitor, and has been shown to be effective, at a low dose, in preventing reocclusion in patients with ICAS-related occlusions (28). In our population, the tirofiban local infusion was used in 40.5% of ICAS-related occlusions. Although the indication for tirofiban infusion was not prespecified in the current retrospective study, it might be used for reocclusion event or the prevention of reocclusion on stenosis. The relative lower rate of reocclusion (24.3%) in our study might be attributed to the preventive tirofiban infusion. On the other hand, considering that in coronary artery occlusive disease both angioplasty and stenting can be used, it has been suggested that these techniques may also be feasible for the treatment of ICAS-related occlusions (30). Although previous negative results from randomized control studies of intracranial stenting for preventing recurrent stroke may discourage from using this procedure (31–33), a very recent study showed that both local tirofiban infusion and angioplasty/stenting treatments were similarly effective and safe in emergent ICAS-related occlusion (34). In another study, permanent stent deployment as a rescue therapy for cases with thrombectomy failure, situations that possibly include substantial number of ICAS-related occlusions, showed better outcomes compared to no deployment (35, 36). When the intracranial angioplasty was optimally performed (residual stenosis < 50%), acute reocclusion rate was less compared to suboptimal cases (residual stenosis over 50%) (2.6 vs. 71.4%). After reviewing these retrospective study results, we concluded that appropriate combination of local antiplatelet infusion and intracranial angioplasty with or without stenting could improve the recanalization and clinical outcomes. There is still, however, a need to further prospectively study this treatment approach in patients with ICAS-related occlusions as it could potentially be used as another rescue treatment option following a Solitaire thrombectomy.

This study has a few limitations. First, the retrospective study design is an inherent limitation. However, ICAS-related occlusions cannot be diagnosed prior to EVT. Additionally, physicians can only see the presentation of intracranial large artery occlusions on baseline angiography, which is not sufficient to distinguish ICAS-related occlusions. To overcome this limitation, a large amount of registry data were collected and matching of baseline variables was performed. We therefore believe that both the reperfusion performance and the safety of Solitaire thrombectomy in the ICAS-related occlusions described here are relevant. Second, although propensity score matching was used, all variables could not be matched; occlusion location was unbalanced even after matching. On the other hand, because of a large sample size difference between the two groups, 2:1 group matching was performed. Possibly due to these two factors, paired statistical tests were not performed in our analyses. Accordingly, we inevitably used independent statistical methods in the matched group comparisons (37). Additionally, our primary endpoint was not a clinical disability scale. Instead, we focused on the feasibility of Solitaire device in ICAS-related occlusions compared to embolic occlusions. The current findings must be cautiously interpreted, especially with respect to the main purpose. Last but not the least, we are unable to suggest what the best primary EVT method is for ICAS-related occlusions based on the results of the current study. Based on previously reported evidence, stent retrievers are supported by sufficient positive trial data as a tool for successful removal of clots in intracranial large artery occlusions (2–5, 10, 38, 39, 5). Therefore, the first step for the treatment of ICAS-related occlusions should be to prove that stent retrievers have comparable performance and are safe compared to the usual embolic occlusions. In the current study, a prototype stent was selected for the evaluation among several similar stent retriever designs. The next step will be to compare both the reperfusion performance and the safety between stent retrieval and clot aspiration methods for the ICAS-related occlusions.

In conclusion, the Solitaire stent was shown to have both similar reperfusion performance and safety profile in the treatment of ICAS-related occlusions compared to embolic occlusions. Nevertheless, cases with minor vessel injuries and/or reocclusion tendencies needed further rescue treatments. Further studies evaluating technical approaches for the treatment of ICAS-related occlusions while addressing aforementioned limitations and issues should be conducted to improve patient outcomes.

## Author Contributions

JSL, S-JL, JH, JC, JY, J-HH, C-HK, Y-WK, D-HK, Y-SK, Y-HH, and S-IS contributed conception and design of the study; JSL, Y-HH, and S-IS organized the database; JSL, S-JL, and JY performed the statistical analysis; JSL wrote the first draft of the manuscript; S-JL and JY wrote sections of the manuscript. All authors contributed to manuscript revision, read and approved the submitted version.

### Conflict of Interest Statement

The authors declare that the research was conducted in the absence of any commercial or financial relationships that could be construed as a potential conflict of interest.
